# Investigation of humans individual differences as predictors of their animal interaction styles, focused on the domestic cat

**DOI:** 10.1038/s41598-022-15194-7

**Published:** 2022-07-15

**Authors:** Lauren R. Finka, Lucia Ripari, Lindsey Quinlan, Camilla Haywood, Jo Puzzo, Amelia Jordan, Jaclyn Tsui, Rachel Foreman-Worsley, Laura Dixon, Marnie L. Brennan

**Affiliations:** 1 Battersea Dogs and Cats Home, Battersea, London, UK; 2 Centre for Evidence-Based Veterinary Medicine, University of Nottingham, Sutton Bonington Campus, Loughborough, UK; 3 Animal, Rural and Environmental Sciences, Nottingham Trent University, Brackenhurst Campus, Southwell, UK; 4 Jeanne Marchig International Centre for Animal Welfare Education, Royal (Dick) School of Veterinary Studies, University of Edinburgh, Midlothian, UK; 5 Animal Behaviour and Welfare, SRUC, Midlothian, UK

**Keywords:** Zoology, Animal behaviour, Psychology, Human behaviour

## Abstract

Humans’ individual differences including their demographics, personality, attitudes and experiences are often associated with important outcomes for the animals they interact with. This is pertinent to companion animals such as cats and dogs, given their social and emotional importance to humans and degree of integration into human society. However, the mechanistic underpinnings and causal relationships that characterise links between human individual differences and companion animal behaviour and wellbeing are not well understood. In this exploratory investigation, we firstly quantified the underlying structure of, and variation in, human’s styles of behaviour during typical human-cat interactions (HCI), focusing on aspects of handling and interaction known to be preferred by cats (i.e. ‘best practice’), and their variation. We then explored the potential significance of various human individual differences as predictors of these HCI styles. Seven separate HCI styles were identified via Principal Component Analysis (PCA) from averaged observations for 119 participants, interacting with sociable domestic cats within a rehoming context. Using General Linear Models (GLMs) and an Information Theoretic (IT) approach, we found these HCI PC components were weakly to strongly predicted by factors including cat-ownership history, participant personality (measured via the Big Five Inventory, or BFI), age, work experience with animals and participants’ subjective ratings of their cat behaviour knowledge. Paradoxically, greater cat ownership experiences and self-assessed cat knowledge were not positively associated with ‘best practice’ styles of HCI, but were instead generally predictive of HCI styles known to be less preferred by cats, as was greater participant age and Neuroticism. These findings have important implications regarding the quality of human-companion animal relationships and dyadic compatibility, in addition to the role of educational interventions and their targeting for optimal efficacy. In the context of animal adoption, these results strengthen the (limited) evidence base for decision making associated with cat-adopter screening and matching. In particular, our results suggest that greater cat ownership experiences and self-reports of cat knowledge might not necessarily convey advantages for cats in the context of HCI.

## Introduction

Humans’ individual differences such as their demographics^1^, personality^2^, attitudes and previous experiences^3^, may have important consequences regarding the behaviour and wellbeing of the companion animals they interact with. However, little is known about the causal mechanisms that underpin these relationships, and how such individual differences may influence real-time human-animal interactions (HAI), and their subsequent impact on animals. Domestic cats and dogs are the most globally popular companion animals, and typically occupy important social niches within human society^4,5^. They may be relied upon as a source of social and emotional support, and are thus likely to engage in regular, potentially socially complex interactions with humans^6,7^. To date, investigations of relationships between humans' characteristics, their interactions with companion animals and potential animal-wellbeing implications are mostly limited to human–dog interactions (HDI) and predominantly within training and formal test contexts. For example, a survey of dog owners reported that being male and suffering from moderate depression was linked with greater use of punitive training methods^8^, while direct observations of dog handlers reported that those scoring lower in ‘Agreeableness’ were more likely to use verbal corrections^9^. Assessments of owner-dog dyad performance during an operational task reported that dyads with owners scoring higher in ‘Neuroticism’ and considering the dog a ‘social supporter’ performed less well^10^. During a test battery^11^, owners scoring lower in ‘Openness’ were also found to score more highly for dog-interaction styles associated with the use of commands. In the same study, owners scoring lower in ‘Conscientiousness’ were reported to score higher for styles associated with petting and praising. In contrast, older owners were reported to score lower for petting and praising styles and also lower for styles associated with affection and enthusiasm^11^.

However, it is uncertain how generalisable these phenomena are to typical unstructured social interactions that frequently occur between humans and companion animals, and whether these dynamics are relevant to other popular species such as cats. Compared to the owner-dog dynamic, owner-cat relationships are often characterised by different owner qualities^12^, attitudes towards, and perceptions of their pets^13,14^, in addition to different animal-owner^15^ and owner-animal attachments^16^. It is therefore possible that the dynamics of HCI, and the factors that moderate them, may vary compared to those evident in HDI. Within the domestic home, relationships between owner personality, gender and the overall structure of human-cat dyadic interactions have been identified^17^. Rates at which owners vocalise and attempt to initiate interactions with their cats have also been linked with aspects of owners’ moods^18,19^. However, across these studies, details of the specific cat-handling styles exhibited by humans during HCI, and their associations with human-individual differences and cat comfort were not included. Therefore, the relevance of such human individual differences to variation in HCI styles in contexts outside of the established cat-owner relationship, or their general implications for cat-wellbeing and cat-human relationships remain unclear.

Tactile interactions with cats are considered to have therapeutic benefits to humans and are increasingly included within interventional contexts to improve human wellbeing^20,21^. However, cats are not considered an inherently social or highly tactile species and may have specific preferences for the ways in which they like to be touched and interacted with^22–25^. Despite this, the common occurrence of human-directed aggression during HCI^26–28^ suggests humans’ understanding of cat behaviour and appropriate styles of HCI may be limited. To address this, in a recent study^29^, we incorporated expert understanding of ‘best practice’ styles of HCI into an educational intervention for humans to use during unstructured social interactions with cats. By encouraging humans to engage in styles of HCI which provided the cat with greater levels of autonomy and also emphasised focusing on the cat’s behaviour and comfort, cats responded with increased human-directed affiliative and positively-valanced behaviour, in addition to decreased rates of human-directed aggression and signs of negative affect. However, while application of the intervention improved humans’ global approaches to HCI, it is unclear what the typical ‘baseline’ or pre-intervention structure of humans’ cat-interactions styles actually look like, the degree to which these may (or may not) contain elements of ‘best practice’, and how this may be mediated by humans’ individual differences such as their personality, demographics and previous experiences with cats.

A better understanding of humans’ individual differences and their relationship to variation in HAI is important and may help to prioritise and adapt educational interventions to better suit the needs of individuals (e.g.^30^), as well as support greater human-animal dyadic compatibility. In an adoption context, this latter aspect is pertinent, given the trends amongst rehoming organisations to prioritise or discriminate against certain adopter characteristics, without a clear evidence base of how such human attributes might translate into real-time benefits for the animals involved^31^. For example, while adopters with greater animal-ownership experiences may be preferred for animals with specific behavioural or HAI requirements, in certain cases, this may lead to higher rather than lower animal relinquishment rates for behaviour-based reasons^32^.

The primary aims of the study were therefore to (1) quantify the underlying structure of, and variation in, humans' styles of behaviour during their typical interactions with domestic cats within an adoption context, and to (2) explore the potential importance of humans’ individual differences as predictors of such variation, and in relation to interaction styles known to be preferred by cats.

## Methods

### Ethical statement

**Figure 1:**
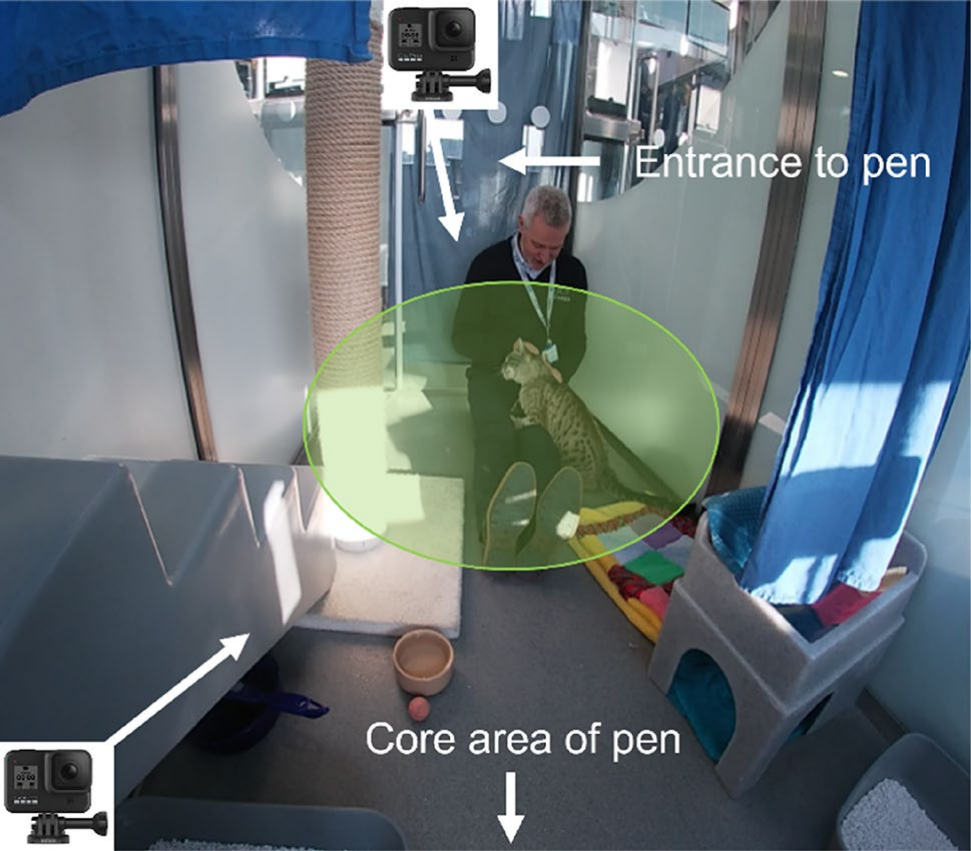
Image captured from GoPro footage depicting experimental set up within an individual cattery pen, including the approximate placement of cameras and their angle of focus (white arrows), the location and typical posture of participants during the test period, and the area within which the cat would be considered as ‘within reach’ of participants (shaded green area, see ethogram in Supplementary File S2 for further details).

Ethical approval for the study was granted by the delegated authority of Nottingham Trent University, Research Ethics Committee ref: ARE192011 and subsequently the delegated authority of University of Nottingham Committee for Animal Research and Ethics ref: 3446 211005. All aspects of experiments were performed in accordance with relevant guidelines and regulations. All personal data provided by participants for the purposes of the study were stored in line with current GDPR guidelines. Informed consent for the publication of Fig. 1 was obtained. Human participants were free to leave the study at any time and gave their informed, written consent to participate. All cats were periodically monitored during data collection by cat welfare experts LF, RFW, CH and JP, in addition to the wider cattery team. Any cats observed showing signs of distress, illness or the potential to injure the participant were immediately removed from the study.

### Participant recruitment and questionnaire completion

Data analysed in this paper formed part of a larger study and dataset collected at Battersea Dogs and Cats Home, UK between January and March of 2020, following the methods outlined in^29^, with the parts relevant to this study also described within this manuscript. Participants were opportunistically recruited via social media and specifically on the basis that they would be interacting with cats for a scientific study. This sampling method was preferred over the recruitment of prospective cat-adopters already visiting the cattery, given the greater likelihood of such individuals trying to present themselves in a more ‘desirable’ way to support their subsequent adoption applications.

Prior to interacting with any cats, participants completed a short questionnaire (see Supplementary File S1 for a copy). The first section of the questionnaire included basic demographic questions (i.e. age category and gender), in addition to several questions about their general cat ownership experiences. Participants were asked to indicate whether they currently lived with cats, if they had any professional or voluntary work experience involving animals, and whether they had ever had any previously negative experiences with cats, each of these variables recorded as a yes/no binary response. Participants were also asked to indicate how many years they had lived with cats and the number of different cats they had lived with over their lifetime, both recorded as continuous variables. Finally, participants were asked to subjectively rate themselves on two 5-point Likert scales for their level of experience with cats (from very experienced to very inexperienced) and knowledge of cat behaviour and body language (from very knowledgeable to very unknowledgeable). Responses to both questions were scored and summed to create a ‘knowledge and experience’ composite variable. The second part of the questionnaire included the 44-item Big Five Inventory (BFI), a widely used, well validated psychometric survey used to assess human personality across key trait dimensions including Agreeableness, Conscientiousness, Extroversion, Neuroticism and Openness^33^.

### Cats included in the study

Participants interacted separately with three unfamiliar cats from a healthy population of predominantly non-pedigree, neutered adult cats (see^29^ and Supplementary File S2 for full details of cat demographics). Video data were initially collected from a total of 114 cats, although contributions from 14 were excluded prior to data analysis, due to either the video coding criteria not being met (n = 6; see further in “Methods” section), early removal from the study for health and welfare reasons (i.e. the cat was scheduled for a veterinary examination or was deemed to be uncomfortable within the cattery on that particular day; n = 4), the cat being rehomed (n = 2), or concerns for participants’ safety, due to the cat behaving overtly aggressively towards the person (n = 2). Cats were housed singly in a cattery pen, containing all their basic resource requirements. Pens measured approximately 2 m × 3 m × 1.5 m. All cats were given time to habituate to the cattery environment prior to study inclusion.

### Methodological controls to limit potential inter and intra-cat human-directed behavioural variability during HCI

A relatively large population of domestic cats were included within the study and participants were exposed to different combinations of study cats over their three HCI tests. In order to reduce the potential noise associated with intra and inter variability in cats’ human directed-behaviour (which might subsequently impact on humans’ cat-directed behaviour during interactions), several controls were implemented:

1. All cats included within the study were assessed by cattery staff members prior to inclusion and deemed suitable to be homed as human companions, meaning they were all considered sufficiently friendly and comfortable around people
2. The temperament of all study cats was subsequently formally assessed using a validated (i.e. convergent, discriminant and predictive validity) questionnaire to confirm the study population was sampled from a sociable, behaviourally homogeneous study population. The questionnaire focused specifically on the traits of human-directed friendliness, fearfulness and frustration reactivity in the context of HCI (see Supplementary File S2 and^29,34^). Average population temperament scores approached the maximum value for friendliness, mid scores for fearfulness and low scores for frustration, with standard deviations relatively conservative in each case (i.e. 16.2/20 ± 2.5, 7.5/13 ± 2.7 and 3.4 ± 1.5 respectively, see Supplementary File S2 and^29^).
3. Participants were required to follow a set of basic protocols (see further in “Methods” section) aimed to reduce cats’ stress and discomfort during HCI, and (to an extent) restrict the way participants were able to physically interact with cats. All cats were therefore presented with a similarly standardised and minimally stressful HCI context, reducing the likelihood for inter-cat variation in high arousal/intensity behaviours (such as human-directed aggression), which could be most likely to impact humans’ subsequent cat-directed responses. Additionally, any cats deemed to be stressed or displaying overt signs of human-directed aggression (see further in “Methods” section) were immediately removed from the study and their observations excluded from analysis.
4. Variability in cats’ human-directed social intent during HCI was further controlled by including only video observations where the cat met a designated threshold for the duration of time they chose to be within sufficient proximity of participants to enable physical HCI to take place (see further in “Methods” section).
5. Participants were also exposed to three separate HCI tests under the same conditions so that their coded behaviours could be subsequently averaged, creating a more general representation of each participant’s cat-directed behaviour. Each coded behaviour was also calculated as a proportion of the total duration of time the cat was within touching distance of the participant. Both approaches aimed to reduce the extent to which the behaviour of a single cat within a single session could impact participants’ overall HCI styles.

### Participant-cat interaction sessions

Participants interacted with three consecutive cats for five minutes each. Participants were instructed to quietly enter the cats’ pen and to sit in the corner nearest to the entrance (away from the cats’ core resting and sleeping area), facing towards the back of the pen. This positioning allowed participants movements to be fully captured via Two GoPro HERO7 cameras, mounted on flexible mini tripods and placed in front and back corners of the pen (see Fig. 1 for an example of the test set up). To balance the need to protect cats’ wellbeing during sessions, limit the risk to participants of being bitten or scratched, but also encourage ‘naturalistic’ styles of interaction, participants were encouraged to interact with the cat as they normally would, with the exception of picking them up. Participants were also asked to remain in their seated position for the duration of the test, which effectively enabled cats to avoid human-interactions if they desired. A curtain was placed over the door of the cats’ pen so that participants would feel less like their actions were being closely scrutinised, with the aim to encourage their more typical styles of HCI. All participants were aware of, and agreed to, their interactions with cats being filmed.

### Testing schedule

Interaction sessions took place during quieter periods within the cattery (i.e. outside of feeding and cleaning times or high visitor numbers). On test days, cats were not provided with opportunities for human interaction except for those occurring during the study sessions or usual feeding and cleaning times. This was to standardise the amount of social interaction to which test cats were exposed, and to avoid possible carry over effects from interactions with staff or volunteers that might then impact the nature of HCI and cats’ human-directed behaviour during test sessions. The majority of cats received a maximum of three, 5-min HCI sessions with different participants, over two consecutive days, with a minimum break of 1.5 h in between each session, again to control for potential carry over effects. To ensure a sufficient number of eligible cats were available each study week, a subset of cats received a second block of test sessions, with a minimum of 1 week in between blocks. The majority of cats (n = 84) thus contributed a maximum of 3 videos to this study, whilst a smaller number contributed a maximum of up to six videos (n = 16).

### Participant behaviour rating

For each session, participants’ cat-directed behaviour was assigned a global rating, based on the degree to which it was generally judged by trained coders to be aligned with ‘best-practice’ principles (i.e. 3 = closely aligned, 2 = somewhat, 1 = not at all), as detailed in^29^). In essence, these principles reflect cat-centric styles of interaction that enable the cat to be the primary initiator and controller of contact, and with the human prioritising the cats’ comfort.

### Participant behaviour coding

**Figure 2:**
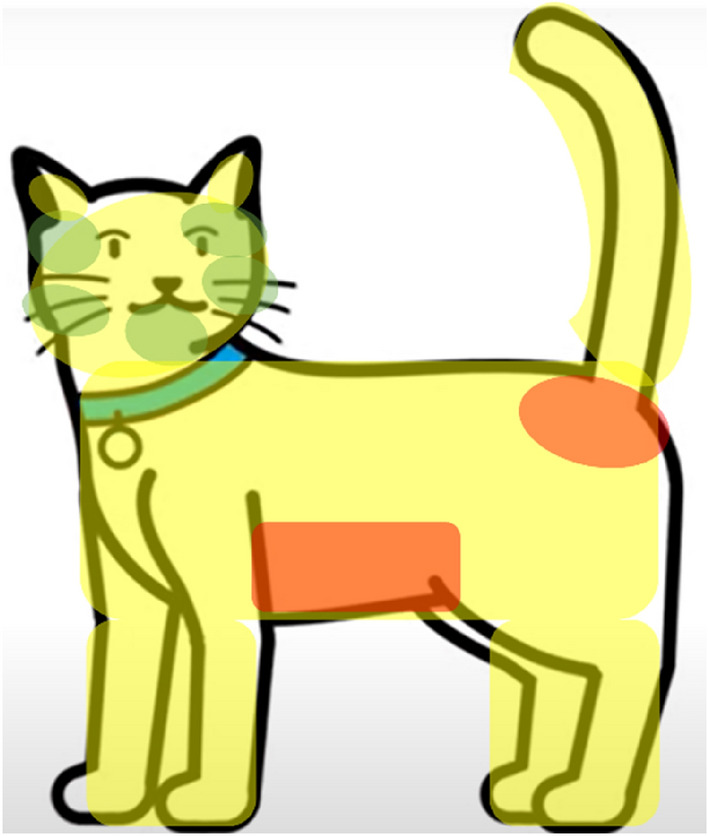
Image depicting the areas coded as ‘Green’ (the base of ears, cheeks and/or under bottom jaw), ‘Red’ (the stomach and base of the tail) and ‘Yellow’ (all other areas), see ethogram in Supplementary File S2 for further details.

Human behaviour was coded across a total of 558 videos by authors LR, AJ and JT in BORIS coding software v. 7.9.8.^35^, using an ethogram comprising of thirteen measures and their operational definitions (see Supplementary File S2). Each behaviour was coded as a duration and/or frequency and was designed to reflect aspects of best practice styles of cat-interaction, as well as their variation. For example, coded measures included frequency of the participant choosing to initiate contact with the cat, as well as frequency of the participant responding to the cats’ request for contact. Other measures included the duration/frequency of participants touching 'green”, “red” and “yellow” areas of the cat (see Fig. 2), with these colours corresponding to areas cats may generally find pleasurable (i.e. the base of ears, cheeks and/or under bottom jaw; green), those they may find unpleasant (i.e. the stomach and/or base of the tail; red) and those where most individual variation in preference occurs (i.e. all other areas; yellow)^24,25^. The ethogram was extensively piloted and modified to ensure it comprised of suitable measures that could be practically coded. Each coder was provided with several training videos and their coding outputs subsequently reviewed by LF. Further support was given until coding competency was reached. For the purposes of inter-rater reliability assessment, 20 videos were then pseudo-randomly selected for coding and rating by all three coders.

### Coding criteria and video eligibility

As participants were instructed to remain seated and not to pursue the cat, the majority of measures could not be coded unless the cat was within touching distance of the person (see Fig. 1 and ethogram in Supplementary Data File S2 for full operational definitions of measures). Thus, to extract meaningful and comparable data across sessions, video eligibility required the cat to be reachable by the participant for ≥ 50% of the session duration (i.e. 150 s). This criteria resulted in some participants having one (n = 45) or two (n = 18) video contributions rather than three.

### Data preparation

A total of twenty frequency and duration-based variables were subsequently extracted. All behaviours were calculated as a proportion of total time the cat was within touching distance of the participant and also sufficiently visible to allow coding. The exception to this was ‘Talks to cat’ which was calculated as a proportion of the total session time (in several instances sessions were slightly shorter than 300 s due to camera malfunction or researcher error).

Behaviour measures and participant ratings were (where possible) averaged across their sessions. Where a participant had only one video contribution, data were taken directly from this observation.

### Statistical analysis

All statistical analyses were performed in R (version 4.0.2)^36^.

### Inter-rater reliability assessment

Intraclass correlation coefficients (ICC2)^37^ were calculated for the ‘best practice’ ratings given to participants as well as all coded behaviours where these occurred frequently enough within the subset of videos to allow for meaningful ICC2 calculations. Measures with ICC2 values of ≥ 0.5^38^ were taken forwards for inclusion in further analysis stages.

### Identification and quantification of variation in human cat-interaction styles

To understand the underlying structure of humans’ behavioural styles during interactions with cats, behaviour data were subjected to a Principal Component analysis (PCA) (via the “psych” package^39^). Bartlett test of sphericity and Kaiser–Meyer–Olkin (KMO) tests were initially performed to confirm sampling adequacy. A Parallel analysis was then undertaken to determine the number of components to retain (via the “paran” package^40^), and a PCA performed using an orthogonal ‘varimax’ rotation^41^, given the small amount of observations (n = 119 averaged behaviour values) relative to behaviour measures included (n = 20). Measures within each of the retained PCs with loadings of ≥ 0.4 were used to support interpretation of each PC in relation to underlying participant ‘interaction styles’. Retained PC scores were then calculated for each participant (using the weighted loadings of measures with loadings of ≥ 0.4).

### Relationship between ‘best practice’ ratings and PC scores

Based on the outcome of the parallel analysis, a total of seven PCs were extracted. To support interpretation of the identified interaction styles (i.e. the separate PCs) in relation to global ‘best practice’ ratings, a general linear model (GLM) with an identity function was performed. Average global ‘best practice’ ratings were included as the response variable and the seven PC scores as the explanatories (as main effects). Non-violation of model assumptions was confirmed via a Shapiro-Wilks normality test and visualisation of the model plots of residuals against fitted values (using the “shapiro.test” and “plot” functions in the core “stats” and “graphics” packages respectively). The Nagelkerke pseudo R squared was performed to assess goodness of fit (using the “nagelkerke” function in the “rcompanion” package^42^).

### Exploring variation in human cat-interaction styles as a function of human individual differences

A series of separate GLMs were created in order to explore the potential for participant individual differences to act as significant predicators of cat-interaction styles. All of the participant demographic variables collected (excluding gender due to its female skew) and personality traits were considered to be of high biological relevance to the main research question and were thus included as explanatory variables. GLMs with logLink functions were created, each with a different participant PC score as the response, and the participant demographic and personality variables as the explanatories (see Table 1 for list of explanatory variables and their descriptive statistics). To avoid the well-highlighted issues associated with stepwise modelling practices^43^, we opted to apply an information theoretic (IT) approach^44^. For each GLM, an initial global model was specified, and then all possible subsets of the explanatory variables simultaneously considered across separate submodels, ranked and subsequently selected based on their Akaike Information Criterion (AIC) values. Model selection was performed using the dredge function (via the MuMIn package^45^), with AIC(c) values calculated to correct for conservative sample sizes relative to the number of explanatory variables. Models with lower AICc were considered a better fit to the data and amongst these, models with AICc deltas (relative difference in AICc values between models) of ≥ 2 were considered to fit the data equally well^46^. Model summaries of ‘best’ models were called and inspected for interpretive purposes. Often, the ‘best’ models can contain several non-significant variables, and several models may fit the data equally well. In this case, the consistency of the presence of a particular explanatory variable, and the relative strength of its association with the PC score (i.e. relative alpha value, sensu^47^) across the higher-ranking models (particularly the top three) was used to understand the level of uncertainty surrounding the predictive relationship between that variable and the relevant PC score. The AICc weight for each model (the proportion of predictive power contained in that model, relative to the total amount of predictive power provided by the full set of models), AICc delta and Nagelkerke pseudo R squared values were used to further support inferences about the predictive strength of the selected models, and subsequently the relative importance of the explanatory variables contained within them.

**Table 1:** Descriptive statistics for the measures collected from participants (n = 119) via the questionnaire (see Supplementary File S1 for a copy), all of which were included as explanatory variables in relevant statistical analysis.

Variable	Mean	SD	Mode	Min score	Max score	Scale range
Years living with cats	22.7712	15.7385	20	0	68	na
Different cats lived with	6.8305	11.6753	3	0	110	na
Knowledge and Experience self-rating composite	8.2542	1.3345	8	2	10	2–10
Agreeableness	35.6949	5.5401	40	21	45	9–45
Extroversion	26.0932	6.4078	26	11	40	8–40
Conscientiousness	34.8305	5.7114	41	17	45	9–45
Openness	37.1186	5.9774	37	18	49	10–50
Neuroticism	23.7119	5.8720	22	8	40	8–40
Age categories: 18–25 = 11 (9%), 26–35 = 36 (30%), 36–45 = 30 (25%), 46–55 = 24 (20%), 56–75 = 18 (15%)						
Live with cats: Yes = 68 (57%), No = 51 (43%)						
Work/voluntary experience with animals: Yes = 49 (41%), No = 70 (59%)						
Previous negative experience with cats: Yes = 27 (23%), No = 92 (77%)						

When applying the dredge function, we were limited to specifying main effects, due to the large amount of explanatory variables included within the global models. Thus, we initially checked for the presence of potentially important multi-way interactions between variables by performing exploratory GLMs on each of the global models, with interactions specified between all explanatory variables (excluding categorical and binomial).

## Results

### Human demographics

The descriptive statistics for the demographic variables collected from participants are presented in Table 1. A total of 120 participants took part in the study, with contributions from one participant subsequently removed due to an incomplete questionnaire. Of the 119 participants included in the data analysis, the vast majority were female (90%). Ages spanned the following ranges: 18–25 (9%), 26–35 (30%), 36–45 (25%), 46–55 (20%), 56–65 (12%) and 66–75 (3%) with these latter two categories collapsed and replaced with the category 56–75 prior to statistical analysis. 57% of participants currently lived with at least one cat and 23% had had a previous negative experience with cats (of any sort). 41% of participants had previous work experience involving animals and details provided described predominantly voluntary roles in rescue and rehoming centres, including animal care and fostering of cats and dogs, although rabbits and horses were also mentioned, in addition to work with zoos and wildlife organisations.

### Inter-rater reliability assessment

All ICC2 values were ≥ 0.5, with most ranging from 0.8 to 0.99, indicating generally very good levels of agreement amongst coders (see Supplementary File S2 for full results) and thus none were excluded from subsequent analyses.

### Human cat-interaction styles and their relevance to ‘best practice’ and its variation

Seven principal components were retained, collectively explaining almost 80% of the total variance, although proportions for individual PCs were modest (i.e. from 16% for PC1 to 7% for PC5). Eigen values ranged from 3.257 (PC1) to 1.438 (PC5) (see Supplementary File S2 for full PCA output).

#### PC1: passive but responds to contact, minimal touching (i.e. ‘Best practice’)

Measures retained for PC1 included various behavioural elements that were anticipated to reflect aspects of ‘best practice’ (see coding ethogram within Supplementary File S2). Retained elements included greater bouts of responding to contact, remaining passive and disengaging from contact, and lower amounts of touching the cat, including lower touching of ‘yellow’ areas. Thus, this PC reflected HCI styles that better allowed the cat (rather than the person) to dictate the nature and quantity of tactile contact.

#### PC2: initiates/disengages contact, touches ‘yellow’ areas

Retained measures included greater bouts of the person initiating and then breaking contact with the cat and touching ‘yellow’ areas. Thus, this PC potentially provided the cat will less choice and control over the nature and frequency of tactile interactions and included touching areas of the cat that were potentially less preferred.

#### PC3: greater contact, touches ‘yellow’ areas, no play

Retained measures were associated with greater durations of actively engaging in contact with the cat (overall and to ‘yellow’ areas) and an absence of initiating play. Similarly to PC2, it is possible this PC provided the cat will less choice and control over the duration of tactile interactions and included touching areas of the cat that were potentially less preferred.

#### PC4: touches ‘green’ areas

Retained measures were exclusively associated with greater durations and frequencies of making contact with ‘green’ areas, those known to be generally preferred by cats.

#### PC5: holds/restrains cat

Retained measures were associated with bouts of holding/restraining the cat, thus removing the cats’ choice and control over the nature of tactile interaction and ability to remove themselves from the interaction, and likely touching the cats’ ‘yellow’ and/or ‘red’ areas in the process.

#### PC6: attempts to engage, touches cat, including ‘yellow’ areas

Retained measures were associated with greater bouts of offering a hand to the cat (thus potentially encouraging interaction in a non-threatening way), and also greater bouts of touching the cat, including ‘yellow’ areas (thus potentially less preferred areas).

#### PC7 touches ‘red’ areas

Retained measures were exclusively associated with greater durations and frequencies of making contact with ‘red’ areas, those known to be generally unpleasant for cats.

### Associations between global ‘best practice’ ratings and PC scores

There was very strong evidence for a positive association between participants’ average best practice ratings and their PC1 scores (mean rating = 1.838936, estimate = 0.31574, standard error = 0.04334, t = 7.286, *p* < 0.0001, pseudo r^2^ = 0.431805), but no significant evidence of relationships between global handling ratings and any of the other PC scores (all *p* > 0.05).

### Humans’ individual differences as predictors of HCI styles

Key results are described below and also summarised in Table 2. For full details of relevant statistical outputs, see Supplementary File S2.

**Table 2:** Summary table including PC components retained, item contents for each PC, their loadings and PC summary in relation to humans’ interactions styles.

Outcome of principal component analysis				Relationship between PC scores and human individual differences (based on multi-model performance)	
PC	Retained measures	Items Loading ≥ 0.4	Interaction style summary	Explanatory variables	Direction and strength of evidence
PC1	**Responds to contact (f)**	0.782		Years living with cats	**
	Touches yellow (f)	− 0.451			
	Physical contact total (d)	− 0.466	Passive but responds to contact, minimal touching (i.e. Best practice')		
	**Remains passive (f)**	0.905			
	**Remains passive (d)**	**0.817**			
	**Disengages, doesn’t retract hand (f)**	**0.598**			
	**Disengages, retracts hand (f)**	**0.409**		Work experience	*
PC2	**Initiates contact (f)**	**0.706**	Initiates/disengages contact and touches ‘yellow' areas	Years living with cats	*
	**Touches yellow (f)**	**0.662**			
	**Disengages, retracts hand (f)**	**0.695**		Extroversion	*
PC3	**Touches yellow (d)**	**0.71**	Greater contact, touches ‘yellow' areas, no play	Different cats lived with	*
	**Physical contact total (d)**	**0.681**			
	Initiates play (f)	− 0.824		Years living with cats	*
	Initiates play (d)	− 0.861		Neuroticism	*
PC4	**Touches red (f)**	**0.952**	Touches ‘red' areas	Knowledge/experience	(approaching ≤ 0.05)
	**Touches red (d)**	**0.964**		Agreeableness	*
PC5	**Holds/restrains cat (f)**	**0.773**	Holds/restrains cat	Age 56–75	***
	**Holds/restrains cat (d)**	**0.8**			
				Neuroticism	(approaching ≤ 0.05)
PC6	**Touches yellow (f)**	**0.461**	Attempts to engage and touches ‘yellow' areas	na	na
	**Physical contact total (f)**	**0.83**			
	**Offers hand (f)**	**0.878**			
PC7	**Touches green (f)**	**0.843**	Touches ‘green' areas	Knowledge/experience	**/***
	**Touches green (d)**	**0.885**			

Items in bold signify positive loadings. PC relationships with individual explanatory variables, direction of effects and strength of evidence (*p < 0.05, **p < 0.01, ***p < 0.001) are also presented. For full details of model performance and outputs from top 3 ranked GLMs for each PC, see Supplementary File S2.

#### Predictors of PC1 scores (passive but responds to contact, minimal touching: ‘Best practice’)

Years living with cats and work experience with animals were the most consistently included variables across the higher-ranking subset models. AICc values were generally very similar across the top-ranking subsets (i.e. AICc deltas did not reach > 2 until the 25th ranked model)**.** The top three models consistently provided strong evidence of a negative relationship between PC1 scores and years living with cats and also weak to moderate evidence of a positive relationship between PC1 scores and animal work experience.

#### Predictors of PC2 scores (initiates/disengages contact, touches ‘yellow’ areas)

Extroversion scores and years living with cats were the variables most consistently included across the higher-ranked sub models. AICc delta between the top and second top model approached the > 2 threshold (i.e. 1.35). The top model provided moderate evidence for positive associations between PC2 and Extroversion scores and also years living with cats, with the second and third models providing weak evidence of the same relationships.

#### Predictors of PC3 scores (greater contact, touches ‘yellow’ areas, no play)

Different cats lived with, years living with cats, and Neuroticism scores were the variables most consistently included across the higher-ranking sub models, for which AICc values were very similar. The top three models consistently provided moderate evidence of positive associations between PC3 scores and different cats lived with and negative associations between PC3 scores, years living with cats and Neuroticism scores.

#### Predictors of PC4 scores (touches ‘red’ areas)

Knowledge/experience and Agreeableness scores were the most consistently included variables across the higher-ranking sub models. AICc values were again very similar across the top three, and these models provided weak to moderate evidence of a negative relationship between Agreeableness scores and PC4 scores, and weak evidence of a positive relationship between knowledge/experience ratings and PC4 scores.

#### Predictors of PC5 scores (holds/restrains cat)

Age and Neuroticism were the most consistently included variables across the higher-ranking sub models. AICc values were very similar across the top three, which consistently provided very strong evidence of a positive relationship between PC5 scores and the age category 56–75. These models also provided weak evidence for a positive relationship between PC5 and Neuroticism scores.

#### Predictors of PC6 scores (attempts to engage, touches cat, including ‘yellow’ areas)

The top-ranking model for PC6 reflected the null model and none of the explanatory variables were consistently included across the higher-ranking sub models. Therefore, no evidence was provided for the presence of meaningful relationships between PC6 scores and any of the explanatory variables.

#### Predictors of PC7 scores (touches ‘green’ areas)

Knowledge/experience rating was the only variable to be consistently included across the higher-ranking sub models. AICc values were very similar across the top three, and these provided strong to very strong evidence of a positive relationship between PC7 scores and knowledge/experience rating.

Across the models for the different PC scores, delta AICcs amongst the higher-ranking models generally suggested that no one model performed exceptionally better that any of the others. Additionally, AICc weights and pseudo r^2^ values were generally relatively low and similar amongst the top performing sub models (see Supplementary File S2). However, in most cases, individual variables demonstrated consistency in their presence (and strength of significance) amongst the higher-ranking sub models, increasing the certainty around their importance as predictors of PC score variation. Collectively, these results would suggest that several of the variables relating to humans’ individual differences are predictive of the identified handling styles, although their effects are likely conservative.

## Discussion

The underlying structure of humans’ behaviour during HCI were quantified and seven separate components relating to variation in interaction styles identified. The component explaining the greatest amount of variation in humans’ behaviour (i.e. PC1) included various elements anticipated to facilitate greater cat autonomy during HCI. Participants’ PC1 scores were also strongly positively associated with global ‘best practice’ ratings, and this was the label subsequently attributed to this PC component.

A series of human characteristics were found to be weakly to very strongly predictive of HCI component scores, although different characteristics were often associated with different aspects of HCI styles. Interestingly, the best predictor of ‘best practice’ PC1 scores was number of years living with cats, although greater years strongly predicated lower scores. Years living with cats was also moderately predictive of higher PC2 scores, reflecting greater bouts of the person (rather than cat) initiating and also disengaging from contact and touching the cat’s ‘yellow’ regions, but also lower PC3 scores, suggesting lower overall duration of contact. Collectively, these associations might reflect the characteristic method of cat-stroking, where a hand is placed at the top of the cat's head and is moved down their back to either the base or tip of their tail (i.e. mostly their ‘yellow’ areas) in a rapid motion, with contact then broken as the person moves their hand back to the top of the cat's head. While a seemingly typical style of human-initiated tactile stimulation of cats, this method of stroking is characteristically quite different to the styles of contact initiated and/or preferred by cats during cat-cat and cat-human interactions. These are often localised to their gland rich areas such as the perioral and temporal regions (i.e. ‘green’ areas)^24,25,48^.

None of the other participant variables relating to cat-ownership experiences including self-rated cat knowledge/experience, currently living with a cat or number of different cats lived with were predictive of best practice HCI styles, although the latter variable was moderately predicative of PC2 scores, representing greater durations of touching ‘yellow’ areas and fewer bouts of initiating play. While subjective self-rated cat knowledge/experience was strongly predictive of touching ‘green’ areas of the cat (i.e. PC7) it was also weakly predictive of touching ‘red’ areas (i.e. PC4). Thus, rather than conveying a greater understanding of the general preferences of cats regarding where they prefer to be touched, higher knowledge/experience self-ratings amongst humans might simply reflect a greater tendency to touch cats across more diverse areas of their body. The lack of positive relationships between ‘best practice’ handling styles, ownership experiences and self-evaluations of knowledge appears consistent with previous literature in domestic dogs, where similar variables were not strongly associated with increased animal empathy or more accurate interpretations of dog behaviour^49,50^.

In contrast to participants’ informal cat ownership experiences and self-rated knowledge, having previous animal-work experience was weakly predictive of higher ‘best practice’ HCI scores (i.e. PC1). This association may be explained by such individuals having greater access to educational materials and training which may enhance their behavioural competency^51,52^, or is perhaps reflective of their having a greater affinity towards animals^53^. However, ‘animal work experience’ in this study represented a diverse range of experience types, with some participants having volunteered with cats, and others with different companion and zoo animals or native wildlife. Thus, more detailed investigation of the potential relationships between humans’ HAI styles, work experience type and species, in addition to their general attitudes towards animals, would be beneficial.

Participant age and personality scores were also predictive of different aspects of their HCI styles, although featured less prominently across the HCI components than the variables associated with their previous ownership experiences. In general, identified relationships appeared congruent with the limited literature in this area. For example, the participant age category of 56–75 was very strongly predictive of PC5 scores, reflecting more frequent and longer bouts of restraining the cat, and thus potentially indicative of more ‘traditional’ or ‘controlling’ styles of HAI, as has been postulated in dogs^11^. Greater cat-restraint amongst older individuals would make sense, given that emphases on animal agency and animals-as-stakeholders during HAI are comparatively recent concepts^54–56^ and thus potentially less-well incorporated into more traditional or historical forms of HAI. PC5 scores were also weakly positively associated with Neuroticism scores, which would fit with the notion of cat-restraint as a form of control, given the broader cross-species associations between human Neuroticism, more controlling and authoritarian interaction styles, and the associated negative outcomes for the recipients^2,8,57,58^. On the other hand, Agreeableness scores were moderately predictive of lower PC4 scores, suggesting a lower tendency to touch ‘red’ areas of cats, and thus potentially a more empathic approach to this aspect of HCI (e.g.^57^). Extroversion scores were also moderately predictive of PC2 scores (suggesting greater bouts of initiating/disengaging from contact and touching ‘yellow’ areas). This would fit with the general notion of extroverts as greater initiators of social interactions^33^, although would appear in contrast to another HCI based study^18^, where owners that reported more extroverted moods were less likely to attempt to initiate contact with their cats.

It is possible that these identified links between human-individual differences and variation in their HCI styles are in part driven by variations in the hedonic value of tactile interactions. For example, while tactile and non-tactile forms of HCI may produce equally pleasant emotional experiences in humans at a population level^59^, females with higher levels of Neuroticism may experience greater positive emotional responses during tactile HCI ^60^. Thus, humans might derive different degrees of pleasure from different styles of HCI, based on their various individual characteristics. In future work, a more detailed assessment of the sequence and temporal patterning of human-initiated versus cat-initiated contact (e.g. via applications of Markovian or time-patterned behavioural analysis^17,61^) would be useful. This may help to further characterise and quantify the specific features of tactile HCI that provide the greatest hedonic value to humans, and the role human individual differences may play (i.e. particularly cat ownership experiences, given their relevance in this study). Differences in the characteristics of human pleasure-linked HCI styles versus those that are pleasurable for cats could also be directly contrasted, and such information used to better understand where conflict during real-time HCI may occur between human-cat dyads. This knowledge could prove very useful in enhancing the efficacy of educational interventions aimed to improve HCI experiences for both parties (e.g.^29,30^).

Participants most frequently rated themselves an eight out of a maximum 10 points for knowledge/experience, indicating relatively high self-ratings within a population than might be expected by chance (e.g.^62^). As participants were self-selecting and their interest in interacting with cats implicit, it is possible that our study population contained individuals with greater-than-average cat behaviour knowledge. This is unlikely however, given that higher knowledge/experience ratings did not predict greater ‘best practice’ HCI scores, but were actually associated with greater touching of ‘red’ areas. Instead, the frequent high subjective self-assessments amongst participants might imply ‘self enhancement’ bias, a phenomenon whereby individuals tend to rate themselves more positively than a normative criterion would predict^62^. The specific nature of the relationships between subjective knowledge/experience ratings and HCI styles might also reflect the Dunning-Kreuger effect^63^ where individuals with some limited or incomplete knowledge on a topic (such as cat behaviour) may overestimate their knowledge or performance, compared to those that have less, or more, actual competency. As such individuals tend to have more limited metacognition about their abilities^64,65^, they may be less receptive to, or at least aware of their need for, interventions aimed to improve competence. Thus, in the context of educational strategies aimed to optimise HCI, individuals that tend to provide higher subjective knowledge/experience self-ratings (in the absence of any formal experiences or training) may require more tailored interventions to navigate these potential cognitive biases. These explanations remain speculative however and require further empirical testing, because objective measures of humans’ behaviour during HCI (as collected during the current study) are only one facet of what could be considered a broader set of cat behaviour skills or competency. Thus, future studies that investigate discrepancies between humans’ subjective self-ratings and additional objective measures of cat behaviour knowledge (such as questions with factually correct/incorrect responses (e.g.^3^), are needed. These may help to better establish whether the phenomenon of ‘self enhancement’ bias, particularly amongst those with limited competency (i.e. the Dunning-Kreuger effect) [e.g.^62,63^] is relevant to human-animal interactions and an animal adoption environment.

Within the context of adoption, animal-adopter pairings that enable positive human-animal interactions likely play a critical role in the quality of the human-animal relationship and reduction of relinquishment risk^66–68^. However, a lack of quality evidence to support various adoption criteria may mean that certain adopter attributes are prioritised or discriminated against, without a solid evidence base to support this decision making^31^. For example, adopter suitability may often be assessed based on an individuals’ previous animal ownership experiences^31^. Particularly in the case of animals that have specific behavioural or handling needs, being an ‘experienced’ owner may be an essential part of their adoption criteria. However, in dogs, the (limited) evidence suggests that such owner-attributes do not necessarily convey advantages to the animals in question^32,69^, with our findings implying similar.

Collectively, our results do not support the notion that greater ownership experiences, as measured via subjective (self-reported) knowledge/experience, numbers of cats lived with, or longer periods of cat-cohabitation, convey advantages for cats during HCI. Thus, it is recommended that such characteristics should not necessarily be considered as reliable indicators of an individuals’ competency during HCI, or their suitability to adopt cats with more specific HCI requirements. Potential adopters that present themselves as very knowledgeable/experienced with cats, but without any formal animal work experience, adopters aged 56+, and those having a longer history of cat ownership, may also benefit most from HCI based educational interventions (e.g.^29^).

While a series of human characteristics were identified as predictors of variation in HCI styles, the strength of these relationships varied (i.e. from weak to very strong), and their effects were generally conservative. These findings would therefore suggest that a broader range of human individual characteristics (and their possible interactions) should be considered during future investigations of human’s interaction styles during unstructured HAI. These differences might include humans’ expectations, attitudes and attachment styles (e.g.^70–73^), their (evidenced) knowledge about animal behaviour and body language, in addition to a range of other educational and socio-cultural factors^53^. However, in applied contexts, and for the purposes of adopter assessment criteria, direct observations of human’s HAI styles(and the subsequent provision of constructive feedback) is likely to offer a more practical and ethical strategy than extensive adopter-screening based on such types of individual characteristics.

This study has several limitations which might hinder the broader generalisability of its findings. HCI were observed within a rehoming centre and between novel cat-human dyads. Although various measures were taken to try to encourage more typical or ‘naturalistic’ styles of HCI within this context (see “Methods” section for details), it is possible that the nature and structure of cat-human dyadic interactions may differ to those occurring in the domestic home, and also to those between familiar cat-human dyads. For example, during standardised HCI observed in the domestic environment, cats were reported to respond more negatively to their owners than to strangers^25^. Additionally, awareness of being filmed during HCI may have motivated participants to behave in a more prosocial or conscientious way [i.e.^74,75^] than they might typically do when interacting with cats in the home. Thus, it is recommended that humans’ HCI styles and their associations with human-individual differences be assessed for their context and temporal stability (i.e. between the rehoming centre and the domestic home, and within the domestic home over time).

Although typical of studies assessing human-cat relationships using convenience sampling methods (e.g.^2,72^), there was also a strong female gender skew amongst recruited participants. Thus, while this bias does not necessarily limit the comparability of our results to related literature, future investigation of possible gender differences in HCI styles are recommended, given the potential influence of gender on the general dyadic patterning of HCI^17^ and the presence of gender-linked personality differences, particularly in western cultures^76^.

Various measures were taken to limit the potential impact of inter-cat variability (and its subsequent influence) on participants’ HCI styles (see “Methods” section for full details). However, as social interactions are fundamentally dynamic in nature, it is likely that aspects of participants’ behaviour during HCI were directly, and potentially differentially, influenced by the behaviour of the cats, with cat human-directed behaviour thus contributing an unknown amount of noise to the data. This is most pertinent to the (small) population of participants where data from only one session, rather than averaged values from multiple sessions, were analysed. In general, however, the impact of such noise would be expected to increase the risk of type II, rather that type I errors [e.g.^77,78^]. Thus, in relation to the factors identified as significant predictors of variability in humans’ HCI styles within this study, we would suggest that conclusions drawn from the results are appropriate, albeit potentially conservative.

However, given the potential for individual variation in cats’ preferences for, and their reactions to, certain aspects of HCI (such as the touching of ‘yellow’ areas^24^), and the potential ambiguity of the measure ‘physical contact total’ in this study, we would suggest certain PCs and their relevance to cat’s individual experiences during HCI be interpreted carefully. For example, PC3 was characterised by greater durations of touching ‘yellow’ areas of the cat and engaging in physical contact overall. For ‘Physical contact total’ to be coded, the participant had to ‘actively engage in physical contact with cat by placing hand, finger(s), face or mouth on any part of cat’. While in some cases, this contact may have represented unwanted or unpleasant tactile stimulation to the cat, in others, participants may have performed more of these behaviours because this type of contact was being actively solicited by the cat.

Useful controls in future studies of this kind might include the quantification of human-directed cat social behaviour and its inclusion as either fixed or random effects within statistical models, or even the use of a life-like ‘cat robot’^79^, in order to completely standardise the behaviour of the cat ‘stimulus’. Markovian or time-patterned behavioural analysis^17,61^ are also recommended to explore HCI from a dynamic perspective. Such approaches may help to tease apart elements of HCI that are predominantly mediated by humans’ individual differences, those mediated by the cat (and their individual differences), and those contingent upon both factors.

## Supplementary Information

Supplementary Information 1.

Supplementary Information 2.

## Data Availability

All data associated with this study and its analysis are included within Supplementary File S2.
