# Supplementary material for: Investigation of humans individual differences as predictors of their animal interaction styles, focused on the domestic cat
Source: Sci Rep. 2022 Jul 15;12:12128. doi: 10.1038/s41598-022-15194-7 (PMC9287547; doi:10.1038/s41598-022-15194-7)
Supplement: Supplementary file 1 — Supplementary Information 1. [file 41598_2022_15194_MOESM1_ESM.docx]

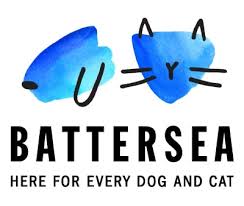

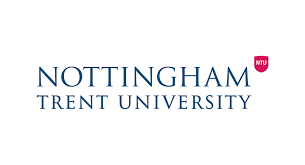


Participant number:

Date:

Time slot:

**Stroke cats for science!**

**Thank you very much for agreeing to help us with our important research.**

Battersea Dogs and Cats Home have teamed up with Nottingham Trent University to undertake an important study to better understand how cats and people interact with each other. We are interested in collecting information about people’s personality, experiences with cats and interactions styles to see how these may relate to the cat-human dynamic. Our aim is to use this information to help improve cat human relationships.

**What will you need to do?**

1. Please **complete this questionnaire**, as truthfully as possible, then place and seal it in the envelope provided. Any information you provide will be confidential and not read by any of the rehoming staff, therefore it will not affect the adoption process if you are intending to rehome a cat.
2. Once completed, you will be taken to **visit six different cats,** each for about 5 minutes. After you have visited three cats (**Part 1**), you will be shown a **short video** before visiting the remaining three (**Part 2**).
3. After each visit, you will be asked a few basic questions about your impression of the cat. Two Go-Pro cameras will be discreetly positioned in each cat’s pen to capture these sessions and will be used for behavioural analysis.
4. Please collect your **Battersea goody bag** as a thank you for taking part!

**We will require about 1.5-2 hours of your time in total, but you are free to leave the study at any time, should you wish.**

All data you provide in relation to this study will be treated as confidential. It will be stored securely and will only be accessible to the research team. No personal information will be shared with third parties. You must be 18 or over to take part in the study. If you decide that you would like your data to be removed from the study and deleted, please contact Dr Lauren Finka [lauren.finka@ntu.ac.uk](mailto:lauren.finka@ntu.ac.uk) or someone at Battersea cattery before 1/4/2020. After this point, all data will be analysed and prepared for publication and therefore cannot be removed. Further information about your personal data privacy rights can be found in the [Privacy Policy](https://eur03.safelinks.protection.outlook.com/?url=https%3A%2F%2Fwww.battersea.org.uk%2F%25E2%2580%258Bcookies-and-privacy-policy&data=01%7C01%7Clauren.finka%40ntu.ac.uk%7Cc079e2b8336948b7f1c708d7992309c4%7C8acbc2c5c8ed42c78169ba438a0dbe2f%7C0&sdata=FOxXnNESPxF9sRKdyzO2oJ9fz5xOkTCfHdlOP5qVIsw%3D&reserved=0): battersea.org.uk/privacy

**Please indicate your consent to participate in the study:**

**I consent to the information I provide in this questionnaire along with the footage collected during cat interactions, to be used for the purposes of research, including potential publication**

**I confirm I am 18 years of age or older**

PTO to complete the survey…..

**Questionnaire:**

**Q1. Please indicate your age:**

18-25

26-35

36-45

46-55

56-65

66-75

76+

Prefer not to say

**Q2. Please indicate your gender:**

Female

Male

Prefer not to say

Prefer to use own term (Please indicate here):

**Q3. Do you currently live with any cats?**

Yes

No

**If you answered yes, please provide details of their ages, sex and breeds**

Info for any additional cats (if owning more than 4):

Age: Sex: M/F? Breed:

Age: Sex: M/F? Breed:

Age: Sex: M/F? Breed:

Age: Sex: M/F? Breed:

**Q4. During your life to date, how many years in total have you lived with cats for?**

Years living with cats:

**Q5. During your life to date, how many different cats in total have you lived with?**

Total number of cats lived with:

**Q6: Do you have any voluntary or professional experience of working with animals?**

Yes

No

If you answered yes, please provide details:

**Q7. How experienced with cats would say you were?**

| Very experienced |
| --- |
| Experienced |
| Neither experienced or inexperienced |
| Inexperienced |
| Very inexperienced |

**Q8. How knowledgeable about cat behaviour and body language would you say you were?**

| Very knowledgeable |  |
| --- | --- |
| Knowledgeable |  |
| Neither knowledgeable or knowledgeable | |
| Unknowledgeable  Very unknowledgeable |  |

**Q9. What do you like most about cats?**

**Q10. Have you previously had any negative experiences with cats?**

| yes |
| --- |
| no |

If you answered yes, please briefly describe these experiences:

**Q11. Please answer these questions about yourself, picking the answer that first comes to you.**

| **I see myself as someone who....** | **Disagree strongly** | **Disagree a little** | **Neither agree nor disagree** | **Agree a little** | **Agree strongly** |
| --- | --- | --- | --- | --- | --- |
| 1. Is talkative | ⃝ | ⃝ | ⃝ | ⃝ | ⃝ |
| 1. Tends to find fault with others | ⃝ | ⃝ | ⃝ | ⃝ | ⃝ |
| 1. Does a thorough job | ⃝ | ⃝ | ⃝ | ⃝ | ⃝ |
| 1. Is depressed, blue | ⃝ | ⃝ | ⃝ | ⃝ | ⃝ |
| 1. Is original, comes up with new ideas | ⃝ | ⃝ | ⃝ | ⃝ | ⃝ |
| 1. Is reserved | ⃝ | ⃝ | ⃝ | ⃝ | ⃝ |
| 1. Is helpful and unselfish with others | ⃝ | ⃝ | ⃝ | ⃝ | ⃝ |
| 1. Can be somewhat careless | ⃝ | ⃝ | ⃝ | ⃝ | ⃝ |
| 1. Is relaxed, handles stress well | ⃝ | ⃝ | ⃝ | ⃝ | ⃝ |
| 1. Is curious about many different things | ⃝ | ⃝ | ⃝ | ⃝ | ⃝ |
| 1. Is full of energy | ⃝ | ⃝ | ⃝ | ⃝ | ⃝ |
| 1. Starts quarrels with others | ⃝ | ⃝ | ⃝ | ⃝ | ⃝ |
| 1. Is a reliable worker | ⃝ | ⃝ | ⃝ | ⃝ | ⃝ |
| 1. Can be tense | ⃝ | ⃝ | ⃝ | ⃝ | ⃝ |
| 1. Is ingenious, a deep thinker | ⃝ | ⃝ | ⃝ | ⃝ | ⃝ |
| 1. Generates a lot of enthusiasm | ⃝ | ⃝ | ⃝ | ⃝ | ⃝ |
| 1. Has a forgiving nature | ⃝ | ⃝ | ⃝ | ⃝ | ⃝ |
| 1. Tends to be disorganised | ⃝ | ⃝ | ⃝ | ⃝ | ⃝ |
| 1. Worries a lot | ⃝ | ⃝ | ⃝ | ⃝ | ⃝ |
| 1. Has an active imagination | ⃝ | ⃝ | ⃝ | ⃝ | ⃝ |
| 1. Tends to be quiet | ⃝ | ⃝ | ⃝ | ⃝ | ⃝ |
| 1. Is generally trusting | ⃝ | ⃝ | ⃝ | ⃝ | ⃝ |
| 1. Tends to be lazy | ⃝ | ⃝ | ⃝ | ⃝ | ⃝ |
| 1. Is emotionally stable, not easily upset | ⃝ | ⃝ | ⃝ | ⃝ | ⃝ |
| 1. Is inventive | ⃝ | ⃝ | ⃝ | ⃝ | ⃝ |
| 1. Has an assertive personality | ⃝ | ⃝ | ⃝ | ⃝ | ⃝ |
| 1. Can be cold and aloof | ⃝ | ⃝ | ⃝ | ⃝ | ⃝ |
| 1. Perseveres until the task is finished | ⃝ | ⃝ | ⃝ | ⃝ | ⃝ |
| 1. Can be moody | ⃝ | ⃝ | ⃝ | ⃝ | ⃝ |
| 1. Values artistic, aesthetic experiences | ⃝ | ⃝ | ⃝ | ⃝ | ⃝ |
| 1. Is sometimes shy, inhibited | ⃝ | ⃝ | ⃝ | ⃝ | ⃝ |
| 1. Is considerate and kind to almost everyone | ⃝ | ⃝ | ⃝ | ⃝ | ⃝ |
| 1. Does things efficiently | ⃝ | ⃝ | ⃝ | ⃝ | ⃝ |
| 1. Remains calm in tense situations | ⃝ | ⃝ | ⃝ | ⃝ | ⃝ |
| 1. Prefers work that is routine | ⃝ | ⃝ | ⃝ | ⃝ | ⃝ |
| 1. Is outgoing, sociable | ⃝ | ⃝ | ⃝ | ⃝ | ⃝ |
| 1. Is sometimes rude to others | ⃝ | ⃝ | ⃝ | ⃝ | ⃝ |
| 1. Makes plans and follows through with them | ⃝ | ⃝ | ⃝ | ⃝ | ⃝ |
| 1. Gets nervous easily | ⃝ | ⃝ | ⃝ | ⃝ | ⃝ |
| 1. Likes to reflect, play with ideas | ⃝ | ⃝ | ⃝ | ⃝ | ⃝ |
| 1. Has few artistic interests | ⃝ | ⃝ | ⃝ | ⃝ | ⃝ |
| 1. Likes to cooperate with others | ⃝ | ⃝ | ⃝ | ⃝ | ⃝ |
| 1. Is easily distracted | ⃝ | ⃝ | ⃝ | ⃝ | ⃝ |
| 1. Is sophisticated in art, music or literature | ⃝ | ⃝ | ⃝ | ⃝ | ⃝ |

**Thank you very much for your time!** Once completed, please place this questionnaire in the envelope provided and hand it back to the researcher or a staff member. **If you would like to receive information about the outcome of this study or hear about opportunities to be involved in future projects, please leave your email address below**

Email address:
